# Supplementary material for: Modulation of DNA Methylation/Demethylation Reactions Induced by Nutraceuticals and Pollutants of Exposome Can Promote a C > T Mutation in the Breast Cancer Predisposing Gene PALB2
Source: Epigenomes. 2022 Sep 30;6(4):32. doi: 10.3390/epigenomes6040032 (PMC9590087; doi:10.3390/epigenomes6040032)
Supplement: Supplementary file 1 [file epigenomes-06-00032-s001.zip › epigenomes-1895540-supplementary.pdf]

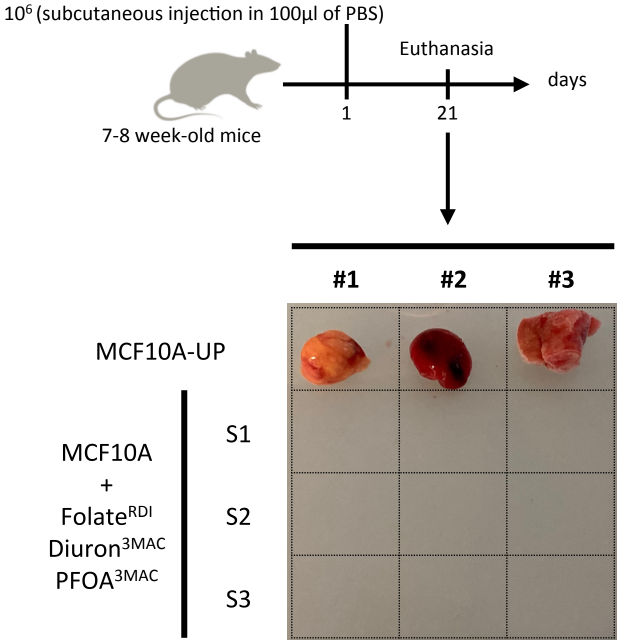

Supplemlentary Figure S1.

Three mice were injected with three batches of MCF10A cells exposed to Folate^{RDI}/Diuron^{3MAC}/PFOA^{3MAC} (S1, S2 and S3). After 21 days, mice were euthanized and the tumors resected. The injection of MCF10A-UP cells is used here as a positive control for mammary tumorigenesis (Pacaud et al. 2014; Duforestel et al. 2019).
